# Exercise-Associated Changes in Body Composition and Metabolic Biomarkers Following an Eight-Week Submaximal Exercise Program in Women Across Different BMI Categories and with Type 2 Diabetes

**DOI:** 10.3390/biomedicines14020473

**Published:** 2026-02-21

**Authors:** Kıvanç Buru, Vedat Çınar, Taner Akbulut, Mehdi Aslan, Meva Ceren Orgun, Fidan Çınar, Orhan Uluçay, Do-Youn Lee

**Affiliations:** 1 Kağızman Vocational School, Management and Organization, Sports Management, Kafkas University, Kars 36000, Türkiye; k.buru@kafkasedu.tr; 2 Faculty of Sport Science, Fırat University, Elazig 23119, Türkiye; cinarvedat@hotmail.com (V.Ç.); mcorgun@firat.edu.tr (M.C.O.); cinarfidantoprak@gmail.com (F.Ç.); 3 School of Physical Education and Sports, Siirt University, Siirt 56100, Türkiye; mhdiasln@hotmail.com; 4 Department of Bioengineering, Faculty of Engineering and Architecture, Kafkas University, Kars 36100, Türkiye; orhanulucay@gmail.com; 5 College of General Education, Kookmin University, Seoul 02707, Republic of Korea

**Keywords:** type 2 diabetes mellitus, submaximal exercise, irisin, myonectin, HIF-1α, insulin resistance, metabolic biomarkers

## Abstract

**Background/Objectives**: This study evaluated exercise-induced changes in body composition and metabolic biomarkers in women across distinct BMI categories and individuals with Type 2 diabetes. **Methods**: In this quasi-experimental study, 40 sedentary women were stratified into five groups (*n* = 8): underweight, normal weight, overweight, obese, and T2DM. The rigorous eight-week supervised program utilized submaximal exercise at 70–85% heart rate reserve, calculated via the Karvonen method and monitored by telemetry. Assessments included anthropometric parameters (BMI, fat mass, visceral fat) and serum biomarkers (irisin, myonectin, HIF-1α, insulin, glucose). Fasting venous samples were collected at baseline and 72 h post-intervention to minimize acute effects, then analyzed using validated ELISA protocols. Statistical data were evaluated using parametric or non-parametric tests with significance set at *p* < 0.05. **Results**: Post-intervention, significant reductions in weight, fat mass, and visceral fat occurred in overweight, obese, and T2DM groups (*p* < 0.05). Muscle mass increased across all cohorts. Fasting insulin and glucose decreased significantly in all except the underweight group, with the most pronounced improvements in T2DM and obese participants. Serum irisin increased significantly across all groups (*p* < 0.05), indicating a universal exercise-induced myokine response. Conversely, myonectin levels decreased significantly only in the normal-weight group, while HIF-1α increased specifically in the T2DM cohort. These findings suggest that baseline BMI and metabolic status are critical determinants of exercise responsiveness, leading to heterogeneous biomarker patterns despite consistent improvements in body composition and basic glycemic regulation. **Conclusions**: An eight-week submaximal program effectively improves body composition and glycemic regulation, though specific biomarker responses are highly dependent on baseline BMI and metabolic status.

## 1. Introduction

There is a growing trend in global mortality, since most fatalities have been attributed to non-communicable diseases, where cardiovascular diseases remain the largest cause of death, with diabetes as a major risk factor in their development. Diabetes quickens atherosclerosis through its harmful effects on the cardiovascular system, thereby raising the incidence of myocardial infarction and other complications. Consequently, controlling diabetes is crucial for reducing the risk of cardiovascular death [1,2]. Diabetes mellitus (DM) is a chronic metabolic disorder caused by either defective insulin production or impaired insulin action, often resulting in severe health outcomes [3]. Diabetes Mellitus is primarily classified into two main types—Type 1 and Type 2 diabetes—with Type 2 diabetes as the most prevalent kind characterized by insulin resistance and subsequent impaired insulin secretion [4]. As per reports released by the WHO, Diabetes is presently one of the world’s fastest-growing public health concerns, a status that it will very likely continue to maintain in the ensuing years [5,6]. This indicates that diabetes extends beyond metabolic imbalance, serving as a systemic risk factor that triggers cardiovascular complications [7].

Age, genetic predisposition, obesity, and lifestyle factors play key roles in the development of T2DM [8]. The strong association between obesity and insulin resistance further amplifies the cardiometabolic burden of diabetes [9]. As a non-pharmacological agent for the prevention and management of diabetes, exercise has been recommended as one of the most effective lifestyle interventions [10]. Regular exercise helps improve glycaemia while also enhancing insulin sensitivity, lowering cardiovascular risks, promoting metabolic health [11]. Exercise-induced release of myokines like irisin and myonectin plays a pivotal role in the regulation of energy metabolism and improved insulin responsiveness [12,13]. Irisin, myonectin, and HIF-1α are some of the prominent biomarkers that have been identified for understanding the relationship between diabetes and physical activity in recent years [14,15]. In the context of T2DM, these biomarkers are increasingly recognized as mediators of metabolic adaptation rather than mere indicators of exercise exposure. Irisin has been linked to enhanced insulin sensitivity and glucose uptake, myonectin to lipid handling and metabolic flexibility, and HIF-1α to hypoxia-related metabolic dysregulation commonly observed in insulin-resistant states. However, findings from exercise interventions remain heterogeneous, and most previous studies have examined either obese or diabetic individuals separately. Direct comparisons of molecular responses to the same structured exercise program across distinct BMI categories together with a T2DM cohort within a single experimental framework are scarce. Therefore, whether baseline BMI and metabolic status differentially modulate exercise-induced changes in these biomarkers remains insufficiently clarified [4,8,12]. Involving metabolic adaptation roles could significantly improve the effectiveness of personalized, exercise-based therapeutic strategies for those with diabetes [16]. Hence, an investigation into the biomarkers influenced by submaximal exercise in different body mass index (BMI) categories of women with Type 2 diabetes should be conducted [17]. By comparing exercise responses among participants with varying BMI classifications and those with diabetes, this study aims to determine in which group the magnitude of effect is most pronounced, thereby identifying the population that benefits most from exercise in diabetes management. Accordingly, it was hypothesized that an eight-week submaximal exercise program would lead to significant improvements in glycemic regulation and body composition, with more pronounced metabolic adaptations in overweight, obese, and T2DM groups compared to metabolically healthier individuals. Furthermore, it was expected that irisin would demonstrate a consistent increase across BMI categories as a universal exercise-responsive myokine, whereas myonectin and HIF-1α responses would exhibit variability depending on baseline BMI and metabolic status. Thus, the study aimed to clarify whether molecular adaptations to submaximal exercise are uniformly distributed or differentially modulated by initial metabolic impairment when evaluated simultaneously across distinct BMI classifications and a T2DM cohort under the same intervention model.

## 2. Materials and Methods

### 2.1. Study Design and Participants

Prior to the study, the statistical power and required minimum sample size were determined using the G*Power software (version 3.1.9.7). The analysis was based on a significance level (α) of 0.05, a test power (1 − β) of 0.80, and a large effect size (f = 0.40). A mixed ANOVA model with four groups and two measurement points (pre-test and post-test) was employed. The correlation among repeated measures was set at 0.50, and the nonsphericity correction (ε) was assumed to be 1.00 [18]. According to these parameters, the power analysis indicated that a minimum of eight participants per group would be statistically sufficient.

**Figure 1:**
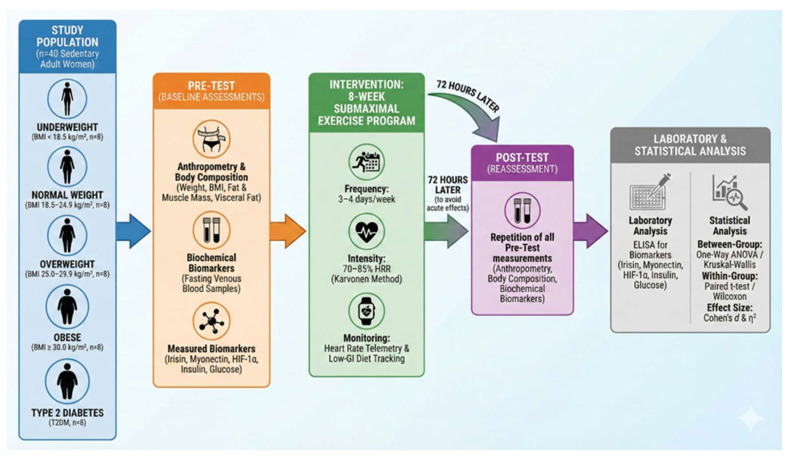
Schematic overview of the experimental study design.

Accordingly, the study was conducted using a quasi-experimental pre–post design with group-based comparisons. The research included voluntary female participants diagnosed with Type 2 Diabetes Mellitus (T2DM) and was carried out at a sports facility located in the city center of Kars, Turkey. A total of 40 participants were enrolled and categorized based on their body mass index (BMI) values as follows: underweight (BMI < 18.5 kg/m^2^, n = 8), normal weight (BMI 18.5–24.9 kg/m^2^, n = 8), overweight (BMI 25.0–29.9 kg/m^2^, n = 8), and obese (BMI ≥ 30.0 kg/m^2^, n = 8). Additionally, individuals diagnosed with T2DM (n = 8) constituted the fifth group. All participants underwent an eight-week submaximal exercise program, and biomarker levels were assessed before and after the intervention (Figure 1).

### 2.2. Inclusion and Exclusion Criteria

Participants included in the study were voluntary women with a basic level of physical fitness who maintained a sedentary lifestyle and agreed to regularly participate in the eight-week exercise program throughout the study period. Individuals with chronic diseases other than diabetes (cardiovascular, respiratory, renal, hepatic, or endocrinological disorders); pregnant women; those requiring regular medication; individuals with a history of psychiatric disorders; and those who had used drugs or supplements that could affect exercise performance or metabolic parameters within the past three months were excluded. Additionally, individuals who had previously participated in similar studies or who had a history of syncope, hematoma, or other adverse reactions related to blood sampling were excluded for safety reasons. Written informed consent was obtained from all participants prior to inclusion in the study. The study protocol was approved by the University Non-Interventional Ethics Committee (Date: 9 January 2024, Decision No: 2024/01-47) and conducted in accordance with ethical principles.

### 2.3. Criteria Anthropometric Measurements

Anthropometric measurements were taken following the approved standardized protocols. Height was recorded with participants standing erect and barefoot using a Holtain stadiometer (accuracy ±0.1 cm). Body weight was recorded using a calibrated Omron BF511T digital scale (accuracy ±0.1 kg) while participants were lightly clothed and shoeless. To improve reliability, the same researcher performed all measurements and repetitions were conducted to minimize potential error. BMI was derived from the weight of the body (kg) divided by the height squared (kg/m^2^).

### 2.4. Blood Sampling and Biochemical Measurements

Blood samples of about 10 mL were withdrawn from all subjects at two different intervals, before and after the exercise program. All the samples were drawn at around 10:00 a.m. from fasting subjects who had been resting for a minimum of 12 h. To prevent a condition in which acute metabolic responses evoked by exercise would bias the results, blood collection after exercise was carried out 72 h after the end of the exercise protocol. At the time of blood collection, sterile tubes were used, and these were immediately centrifuged at 3000 rpm for 10 min to obtain the serum. Care was taken to avoid hemolysis when isolating the serum samples, which were stored in a deep freezer at −80 °C before analysis.

The serum levels of irisin, myonectin, insulin, glucose, and hypoxia-inducible factor-1 alpha (HIF-1α) in different groups were detected using commercial ELISA kits that were validated according to the manufacturers’ protocol. All biochemical analyses were performed in duplicate samples, which were then read at 450 nm using the Thermo Scientific Multiskan GO Microplate (Waltham, Massachusetts, ABD) reader for absorbance. The standards were then established using the result obtained from the study. The kits used were verified for high sensitivity and acceptable reproducibility (intra-assay and inter-assay coefficient of variation, CV < 10%). Measurements were expressed as follows: irisin (Reed Biotech Ltd., Q8NAU1, Wuhan, China) and myonectin (Reed Biotech Ltd., D4AB34, Wuhan, China) in ng/mL; HIF-1α (Reed Biotech Ltd., Q16665, Wuhan, China) in pg/mL; insulin (Reed Biotech Ltd., PO1308) in µIU/mL; and glucose (Reed Biotech Ltd., P11021, Wuhan, China) in mg/dL.

### 2.5. Exercise Protocol

Before the commencement of the study, all participants were informed about the purpose, procedures, and potential risks of the research, and written consent was obtained. A personalized submaximal exercise program was administered to sedentary women in accordance with the American College of Sports Medicine (ACSM) guidelines [19]. Maximum heart rate (MHR) was estimated using the age-predicted formula (220 − age) and was used solely as a component of the Karvonen equation. Exercise intensity was determined exclusively based on the Karvonen method, targeting 70–85% of heart rate reserve (HRR), and the program was conducted 3–4 days per week for eight weeks. Prior to the intervention, a one-week preparatory phase was implemented to minimize the risk of injury and facilitate physiological adaptation. The exercise program consisted of resistance and cardiovascular activities engaging major muscle groups of both the upper and lower extremities, with training intensity progressively adjusted on a weekly basis (Table 1). Heart rate was continuously monitored during each session using Polar heart rate monitors, and resting heart rate was measured each morning upon waking with Polar Ignite GPS watches and reported to the researcher. Target training heart rates were recalculated weekly to account for physiological adaptations, and participants were warned whenever heart rate values exceeded the predefined HRR-based target zones [20]. All sessions were supervised by certified trainers, and participant adherence as well as perceived training tolerance were continuously monitored throughout the intervention period.

### 2.6. Diet Monitoring and Nutritional Program

To minimize potential confounding effects of dietary variability and to ensure homogeneity across groups, participants received individualized nutritional guidance based on estimated daily energy requirements. To maintain dietary standardization, a registered dietitian provided a one-hour nutritional education session to all participants before the intervention. Basal metabolic rate (BMR) was calculated using the Harris–Benedict equation. Total daily energy expenditure (TDEE) was then estimated by multiplying BMR by a physical activity level (PAL) coefficient appropriate for sedentary individuals (PAL = 1.4–1.6), consistent with established nutritional assessment guidelines. A balanced macronutrient distribution (approximately 50–55% carbohydrates, 12–15% protein, and 25–30% fat) was recommended in accordance with national dietary guidelines [21,22]. Daily fluid requirements were determined individually using a body weight–based calculation (approximately 30–35 mL per kg body weight), in line with national nutritional recommendations. Participants were advised to maintain adequate hydration throughout the intervention period [23,24]. No weight-loss-oriented or strict caloric-restriction protocol was imposed. Participants were instructed to maintain consistent dietary patterns throughout the eight-week intervention period.

Twenty-four-hour food consumption records were obtained to monitor dietary intake, and portion sizes were estimated using a standardized food photograph catalogue. Energy and nutrient intake were analyzed using the Computer-Assisted Nutrition Program (BEBIS 9.0). The primary purpose of dietary monitoring was to maintain nutritional consistency among participants and reduce major dietary fluctuations, thereby allowing a clearer evaluation of exercise-induced physiological and molecular adaptations [25,26].

### 2.7. Statistical Analysis

The data obtained in the study were analyzed using IBM SPSS Statistics 25.0 software. All data are presented as mean ± standard deviation. Distributional assumptions were evaluated using skewness and kurtosis values (within the ±2 range) together with formal normality tests [27]. According to distribution characteristics, between-group differences across BMI categories were examined using one-way analysis of variance (ANOVA) for normally distributed variables or the Kruskal–Wallis test for non-normally distributed variables, with results reported as F or χ^2^KW statistics, respectively. Significant main effects were followed by post hoc analyses to identify pairwise group differences, using the Tukey HSD test for normally distributed variables and Dunn’s post hoc test with Bonferroni correction for non-normally distributed variables. Within-group pre–post changes were assessed using the Paired-Samples t-test for normally distributed data or the Wilcoxon signed-rank test for non-normally distributed data, with test statistics reported as t or z values. Effect sizes for between-group comparisons were quantified using eta squared (η^2^) and interpreted as small (≈0.01), medium (≈0.06), or large (≥0.14), while Cohen’s d was calculated to assess the magnitude of within-group changes and interpreted as small (≈0.20), medium (≈0.50), or large (≥0.80). Statistical significance was set at *p* < 0.05 [28].

## 3. Results

Between-group comparisons revealed significant differences across BMI categories for all anthropometric and body composition variables at both pre-test and post-test measurements. For BMI, a significant group effect was observed at pre-test (F = 43.64, *p* < 0.001, η^2^ = 0.83) and post-test (F = 32.21, *p* < 0.001, η^2^ = 0.78), with post hoc analyses indicating higher BMI values in the diabetes group compared with the underweight and normal-weight groups (a > b, c). Within-group analyses demonstrated significant pre–post reductions in BMI for the diabetes (z = 2.527, *p* = 0.012, Cohen’s d = 1.797), overweight (t = 9.924, *p* < 0.001, d = 3.509), and obese groups (t = 21.466, *p* < 0.001, d = 7.589), whereas no significant change was observed in the normal-weight group (*p* > 0.05) (Table 2).

For body weight, significant between-group differences were found at pre-test (F = 42.41, *p* < 0.001, η^2^ = 0.82) and post-test (F = 31.32, *p* < 0.001, η^2^ = 0.78), with the diabetes group exhibiting higher values than the underweight and normal-weight groups (a > b, c). Within-group comparisons indicated significant reductions in body weight in the diabetes (t = 6.546, *p* < 0.001, d = 2.314), normal-weight (t = 2.737, *p* = 0.029, d = 0.968), overweight (t = 10.563, *p* < 0.001, d = 3.734), and obese groups (z = 2.521, *p* = 0.012, d = 5.220), while the underweight group showed a significant increase (t = 5.459, *p* = 0.001, d = −1.930). Significant group effects were also observed for fat mass at pre-test (F = 45.20, *p* < 0.001, η^2^ = 0.83) and post-test (F = 45.42, *p* < 0.001, η^2^ = 0.83), with higher values in the diabetes group compared with the underweight, normal-weight, and overweight groups (a > b, c, d). Within-group analyses revealed significant reductions in fat mass across all groups (*p* < 0.05), with large effect sizes (Cohen’s d range: 1.97–4.17) (Table 2).

For muscle mass, significant between-group differences were found at pre-test (F = 13.06, *p* < 0.001, η^2^ = 0.59) and post-test (F = 6.96, *p* < 0.001, η^2^ = 0.44). Post hoc comparisons indicated lower muscle mass in the diabetes group compared with the underweight, normal-weight, and overweight groups (a < b, c, d). Within-group comparisons demonstrated significant increases in muscle mass in all groups (*p* < 0.05), with large effect sizes (Cohen’s d range: −2.66 to −5.28) (Table 2).

Regarding visceral fat level, significant group differences were observed at pre-test (F = 27.65, η^2^ = 0.76) and post-test (F = 32.85, η^2^ = 0.79). Post hoc analyses indicated higher visceral fat levels in the diabetes group compared with the underweight and normal-weight groups at pre-test (a > b, c), and compared with the underweight, normal-weight, and overweight groups at post-test (a > b, c, d). Within-group reductions were significant in all groups except the underweight group (*p* = 0.059) (Table 2).

Between-group comparisons revealed no significant differences in myonectin levels across BMI categories at either pre-test (χ^2^KW = 0.623, *p* = 0.961, η^2^ = 0.02) or post-test (χ^2^KW = 4.617, *p* = 0.329, η^2^ = 0.17). Within-group analyses indicated a significant pre–post change only in the normal-weight group (t = 2.651, *p* = 0.033, Cohen’s d = 0.94), whereas no significant changes were observed in the other groups (*p* > 0.05) (Table 3).

For HIF-1α, no significant between-group differences were observed at pre-test (χ^2^KW = 2.544, *p* = 0.637, η^2^ = 0.07). However, a significant group effect emerged at post-test (F = 2.656, *p* = 0.049, η^2^ = 0.23), with post hoc analysis indicating higher values in the diabetes group compared with the underweight group (a > b). Within-group comparisons showed no significant pre–post changes across groups (*p* > 0.05) (Table 3).

Regarding irisin, no significant between-group differences were found at pre-test (F = 0.608, *p* = 0.660, η^2^ = 0.06), whereas a significant group effect was observed at post-test (F = 2.581, *p* = 0.050, η^2^ = 0.22), with higher values in the diabetes group compared with the underweight and normal-weight groups (a > b, c). Within-group analyses demonstrated significant increases in irisin levels across all groups (*p* < 0.05), with large effect sizes (Cohen’s d range: 1.21–4.80) (Table 3).

Significant between-group differences were observed for insulin levels at both pre-test (χ^2^KW = 12.773, *p* = 0.012, η^2^ = 0.29) and post-test (χ^2^KW = 15.043, *p* = 0.005, η^2^ = 0.26). Post hoc analyses showed higher insulin levels in the diabetes group compared with all other groups at pre-test (a > b, c, d, e), and compared with the underweight, normal-weight, and overweight groups at post-test (a > b, c, d). Within-group analyses revealed significant reductions in insulin levels in the diabetes, normal-weight, overweight, and obese groups (*p* < 0.05), while no significant change was observed in the underweight group (*p* = 0.126) (Table 3).

For glucose, significant between-group differences were detected at pre-test (F = 6.075, *p* = 0.001, η^2^ = 0.41) and post-test (F = 5.713, *p* = 0.001, η^2^ = 0.39). Post hoc comparisons indicated higher glucose levels in the diabetes group compared with all other groups at pre-test (a > b, c, d, e) and compared with the underweight, normal-weight, and overweight groups at post-test (a > b, c, d). Within-group comparisons demonstrated significant reductions in glucose levels in the diabetes, normal-weight, overweight, and obese groups (*p* < 0.05), whereas the change in the underweight group was not statistically significant (*p* = 0.092) (Table 3).

## 4. Discussion

Following the eight-week submaximal exercise program implemented within the scope of this study, changes were observed in participants’ anthropometric measurements and metabolic biomarkers, which varied according to their initial body mass index (BMI) categories. In terms of body composition, a decrease in BMI, total body weight, and visceral adiposity levels was detected in the diabetic, obese, and overweight groups, while a decrease in fat mass and an increase in muscle mass were observed in all groups. Unlike the other groups, an increase in body weight was observed in the underweight group, while no significant change was found in the BMI of the normal-weight group. At the level of metabolic parameters, insulin and glucose values decreased in the diabetic, obese, overweight, and normal-weight groups, whereas no significant change was observed in the underweight group. Regarding biomarkers, irisin showed an increase in all groups, while a decrease in myonectin was seen only in the normal-weight group. The increase in HIF-1α was statistically significant only in the diabetic group.

In our study, the findings regarding body composition are broadly consistent with existing literature showing that regular submaximal exercise optimizes body weight and fat mass and triggers skeletal muscle hypertrophy, especially in populations at metabolic risk [29,30,31]. For example, in a study conducted by Dupuit et al. (2020) on overweight/obese postmenopausal women, it was reported that a 12-week submaximal exercise program provided significant improvements in body weight, total fat mass, and waist circumference, but produced more limited adaptations in terms of abdominal and visceral adipose tissue and muscle mass increase [32]. Similarly, it has been reported that submaximal exercise programs applied 3–5 days per week for 30–60 min in overweight and obese individuals produce clinically significant reductions in total fat mass and abdominal adiposity [33]. In a controlled study conducted on obese men, Ross et al. (2000) compared the effects of equivalent weight loss achieved through diet and exercise on body composition. They revealed that weight loss achieved with submaximal aerobic exercise was associated with a more pronounced reduction in visceral adipose tissue and better preservation of fat-free body mass compared to diet [34]. These results suggest that submaximal exercise provides an effective stimulus, particularly in reducing abdominal and visceral adiposity, but increases in muscle mass remain limited and secondary. These findings indicate that submaximal exercise provides positive effects, particularly on general body composition and metabolic risk indicators, but shows a more limited effect in terms of regional fat distribution and significant skeletal muscle hypertrophy. In this context, it is thought that the higher effect sizes detected in the body weight, fat mass, and BMI variables in the current study are due to submaximal exercise predominantly activating aerobic metabolic pathways and increasing fat oxidation, while the lower effect sizes for muscle mass are related to the limited stimulation of mechanical load and tension levels required for hypertrophic adaptations by submaximal exercise. This finding appears consistent with previous studies reporting that the effects of submaximal exercise on adiposity and weight control are more pronounced compared to the increase in muscle mass [35,36]. This situation is thought to result from submaximal exercise causing weight and fat mass loss by increasing long-term energy expenditure to create a negative energy balance, making fat oxidation the dominant metabolic pathway, and particularly supporting catecholamine-mediated lipolysis [37]. On the other hand, it is thought that submaximal exercise can create limited but significant increases in muscle mass by positively affecting muscle protein turnover through repetitive muscle activation, maintenance of motor unit recruitment, and increasing exercise-induced myokine release; however, significant hypertrophic adaptations remain limited because the applied mechanical load and tension levels are lower compared to high-intensity or resistance-based exercises [38,39].

In addition to the findings regarding body composition, when biochemical adaptations related to submaximal exercise are examined, it is also seen that changes in metabolic biomarkers differ according to the participants’ initial BMI levels and metabolic profiles. In this context, while the increase in irisin levels in all groups was found remarkable, it was determined that myonectin and HIF-1α responses exhibited a group-specific distribution and the improvement in insulin and glucose levels was more pronounced, especially in the diabetic and obese groups. These findings show that submaximal exercise is not limited to structural changes but also creates adaptive responses that differ at the metabolic and molecular levels. Consistent with these findings, previous studies have reported that biochemical responses to submaximal exercise exhibit different adaptation patterns in line with individuals’ initial metabolic profiles [40,41]. Among these biochemical responses, irisin, which stands out as an exercise-sensitive myokine, particularly attracts attention in the literature for understanding metabolic adaptations to submaximal exercise. The main reason for this is that irisin is released via FNDC5 expression with skeletal muscle contraction, is sensitive to exercise duration and the continuity of muscle activity, and its circulating levels consistently increase, especially in moderate-intensity, long-term aerobic exercises. Furthermore, the fact that irisin supports the browning response in adipose tissue, increases energy expenditure, and is associated with the improvement of glucose homeostasis and insulin sensitivity makes it an important biomarker reflecting the metabolic effects of exercise [42,43].

Accordingly, the significant increase in irisin levels in all BMI and metabolic groups in the current study is evaluated as a finding consistent with previous studies reporting that submaximal exercise consistently stimulates the irisin response. For example, in a study examining the relationship between 12-week exercise training and irisin and glucose metabolism in middle-aged, overweight, and obese men with impaired glucose regulation, plasma irisin levels were reported to increase significantly [44]. Similarly, in the study conducted by Soori et al. (2016) on obese men, it was reported that a 10-week submaximal aerobic exercise program (3 days a week, 30–45 min) significantly increased serum irisin levels compared to the control group [45]. In this framework, the irisin increase observed in individuals with different BMI and metabolic profiles in the current study supports previous human studies reporting that submaximal and continuous aerobic exercises consistently stimulate the irisin response independent of individuals’ metabolic status [46,47]. Additionally, positive changes similar to the irisin response were observed in glucose and insulin levels; however, these changes were not found statistically significant in the underweight group. In contrast, the more pronounced exercise-induced glucose and insulin responses in overweight, obese, and diabetic individuals show that the regulatory effect of submaximal exercise is stronger in cases where metabolic impairment is more advanced. This situation is directly consistent with the findings of de Lannoy et al. (2017), which were carried out in individuals with abdominal obesity and showed that glucose and insulin responses to exercise differ depending on the initial metabolic state [41]. In the study in question, it was reported that glycemic improvements after submaximal and aerobic exercise interventions occurred significantly only in individuals with initial insulin resistance and glucose intolerance, whereas exercise-induced glucose and insulin changes remained limited in metabolically healthier individuals.

Indeed, Barwell et al. (2008), in their study comparing women at risk for insulin resistance, such as first-degree relatives of individuals with type 2 diabetes, with healthy controls with no history of diabetes, reported that despite the same exercise training, the increase in insulin sensitivity was significant only in the metabolic risk group [48]. Similarly, in a study comparing healthy (lean and normal glucose tolerance), obese, and type 2 diabetic individuals, it was reported that insulin-mediated metabolic responses differed significantly depending on the degree of metabolic impairment. In the study, it was shown that insulin sensitivity and metabolic flexibility were largely preserved in healthy individuals, whereas insulin-mediated glucose utilization and metabolic suppression were significantly impaired in obese and especially type 2 diabetic individuals. Researchers emphasized that although exercise interventions create regulatory effects on metabolic parameters, this effect becomes more pronounced as the level of initial metabolic impairment increases [49]. In fact, it has been reported in the literature that glycemic and insulin responses to exercise show significant variability among individuals, and this variability is largely determined by the initial level of glucose intolerance and insulin resistance [41,48]. Especially in individuals with more advanced metabolic impairment, exercise-induced improvements are more frequently observed, while in normoglycemic and thin individuals, these responses mostly remain within the limits of measurement variation. These findings clearly demonstrate that the effect of submaximal exercise on glucose homeostasis strengthens as the metabolic risk level increases and that biochemical adaptations to exercise are shaped depending on the individual’s initial metabolic profile, showing consistency with findings reported in the literature.

However, it is noteworthy that the change in HIF-1α levels, contrary to body composition and other metabolic biomarkers, was statistically significant only in the diabetic group. When the current literature is examined, it is seen that there are a limited number of studies directly comparing the HIF-1α response after submaximal exercise between healthy and metabolically impaired individuals that clearly report a group-specific increase; in this context, the current finding does not have a one-to-one equivalent in the literature. Nevertheless, De Groote et al. (2021), in their study examining the effects of submaximal exercise on glucose tolerance and hypoxia-sensitive signaling pathways at the muscle level under normoxic and hypoxic conditions in healthy and prediabetic individuals, did not detect a significant change in HIF-1α protein levels despite activation of exercise-induced metabolic signals, but observed that HIF-1α levels were higher in prediabetic individuals [50]. In this regard, in the comprehensive review study conducted by Gabryelska et al. (2020), it is emphasized that HIF-1α is often increased or abnormally regulated, especially in metabolic environments characterized by insulin resistance and type 2 diabetes, and this phenomenon is closely related to glycolysis becoming dominant, suppression of oxidative metabolism, and worsening insulin resistance [51]. Within this framework, the significant change in the HIF-1α response only in the diabetic group in the current study suggests that diabetes-specific metabolic and oxygenation disorders may have activated hypoxia-sensitive signaling pathways more markedly during submaximal exercise, indicating that HIF-1α may be a biomarker sensitive to the individual’s initial metabolic state in terms of metabolic adaptations to exercise. Mechanistically, chronic hyperglycemia and insulin resistance are associated with mitochondrial dysfunction, reduced oxidative phosphorylation efficiency, and increased reactive oxygen species production, all of which may promote stabilization of HIF-1α even under normoxic conditions [50]. During submaximal exercise, the increased metabolic demand in diabetic individuals may further amplify this pre-existing hypoxia-sensitive signaling, resulting in a measurable elevation of HIF-1α. This pattern suggests that HIF-1α may function not merely as a hypoxia marker but also as an indicator of altered mitochondrial and redox homeostasis in metabolically compromised states.

Similar to HIF-1α, it was observed that the myonectin response to submaximal exercise was not homogeneous among groups and showed a statistically significant change only in a specific group. This suggests that exercise-induced myokine responses are not uniform; on the contrary, the metabolic effects of exercise can create group-specific adaptations depending on the individuals’ initial metabolic profile and require the role of myonectin in exercise-induced metabolic regulation to be handled within this framework. Indeed, when the literature is examined, it is seen that there is no consistency among studies regarding the direction and magnitude of exercise-induced myonectin responses: an increase is reported in some studies, no change in others, or opposite responses are reported [52]. For example, in the study of Lim et al. (2012), it was reported that circulating myonectin levels decreased statistically significantly after 10 weeks of aerobic exercise in both healthy young women and elderly overweight women [53]. That is, in this study, exercise led to a decrease in myonectin, not an increase, in both groups [53]. On the other hand, in the study of Choi et al. (2013), it was reported that circulating myonectin levels increased statistically significantly after a 12-week combined (aerobic + resistance) exercise program applied in obese women [54]. In light of this information, our findings that biomarker responses to exercise do not exhibit a homogeneous change in all groups and that myokine responses in particular can show group-specific differences sensitive to the metabolic context are consistent with the literature. Myonectin is closely linked to systemic lipid trafficking and fatty acid uptake, and its regulation appears dependent on substrate availability and insulin-mediated metabolic flux. In metabolically balanced individuals, improved lipid oxidation efficiency induced by exercise may reduce the requirement for elevated myonectin signaling, potentially explaining the decrease observed in the normal-weight group. In contrast, persistent alterations in lipid turnover and inflammatory tone in metabolically impaired states may attenuate or dysregulate this adaptive response [52,53]. These findings suggest that myonectin dynamics may reflect differences in skeletal muscle–adipose communication and lipid partitioning efficiency rather than a uniform exercise-driven increase. From a clinical perspective, the present findings suggest that submaximal exercise programs may yield differential metabolic and molecular benefits depending on baseline BMI and metabolic status. While improvements in glycemic regulation appear more pronounced in overweight, obese, and T2DM individuals, biomarker responses such as myonectin and HIF-1α may reflect BMI-dependent molecular adaptations. Therefore, exercise prescription in women with metabolic risk should consider initial metabolic profile rather than adopting a uniform approach. These results support the concept of individualized exercise strategies aimed at optimizing metabolic and molecular outcomes in T2DM management [40,41,55].

## 5. Conclusions

In this study, it has been shown that an eight-week submaximal exercise program leads to significant changes in body composition and metabolic biomarkers in individuals with different body mass index categories. Following the submaximal exercise intervention, an improvement in glucose and insulin levels was observed along with a decrease in body weight, fat mass, and visceral adiposity, especially in diabetic, obese, and overweight individuals. In contrast, it was determined that these biochemical changes remained at a more limited level in metabolically healthier and thin individuals. When evaluated in terms of biomarkers, while the increase in irisin levels in all groups reveals a common myokine response to submaximal exercise, the fact that changes in HIF-1α and myonectin levels were significant only in certain participant groups shows that molecular responses to exercise can differ according to the individuals’ initial metabolic characteristics. These results demonstrate that the effects of submaximal exercise on biomarkers associated with body composition, glucose, and insulin metabolism are not uniform, and that the initial metabolic status of individuals is an important determinant in shaping the physiological and biochemical responses to exercise. In practical terms, exercise prescription should be structured according to the individual’s baseline metabolic status. Submaximal aerobic programs performed within heart rate reserve–based target zones may be adjusted in intensity and volume depending on the degree of metabolic impairment and glycemic regulation. Individuals with greater metabolic dysregulation may require carefully monitored progressive protocols to optimize glycemic and molecular responses, whereas those with relatively stable metabolic profiles may tolerate gradual increases in training load to further stimulate adaptive responses. Therefore, initial metabolic assessment should guide the selection of exercise intensity, volume, and progression strategy rather than applying a uniform intervention model.

## 6. Strength, Weakness, and Recommendation

This study is strengthened by its comparative design, which evaluated women across different BMI categories together with individuals with Type 2 diabetes under the same standardized submaximal exercise protocol, allowing the examination of exercise-associated adaptations according to baseline metabolic status. The combined analysis of anthropometric outcomes and exercise-related metabolic biomarkers (irisin, myonectin, and HIF-1α), alongside heart rate reserve–based intensity control and the reporting of effect sizes, provides a comprehensive view of both physiological and molecular responses to exercise. Nevertheless, the relatively small sample size, the absence of a non-exercising control group, and the short intervention duration limit causal inference and generalizability, and may have contributed to inflated within-group effect size estimates. Although dietary intake was carefully monitored and standardized according to individualized energy requirements, residual nutritional variability cannot be entirely excluded. Future studies employing larger randomized controlled designs, longer intervention periods, stricter nutritional standardization, and analyses focused on inter-individual variability and baseline metabolic status are warranted to support precision-based exercise prescriptions in metabolic disease management.

## Figures and Tables

**Table 1:** Schematic representation of the eight-week submaximal exercise protocol designed according to the Karvonen method. The program consisted of warm-up, cardio, resistance, and core exercises, with weekly volume progressively increased while maintaining muscle group variety. Core muscles were trained in a balanced and functional manner, and exercise progression was achieved through incremental load increases and exercise variation. Rest intervals were adjusted based on individual adaptation levels.

Week	Warm-Up—Cardio (25 min)	Resistance Exercises (3 × 10 Repetitions)	Core Exercises	Intensity Level
**1st Week**	5 min stretching 5 min cycling 10 min treadmill 5 min elliptical	1. Squat 2. Leg Curl 3. Front Dumbbell Raise 4. Biceps Curl 5. Lat Pull Down	Crunches (3 × 15) Plank (3 × 20 s)	Beginner
**2nd Week**	5 min stretching 5 min cycling 10 min treadmill 5 min elliptical	1. Leg Extension 2. Side Dumbbell Raise 3. Push Down 4. Hip Thrust 5. Deadlift (light)	Reverse Crunch (3 × 12) Side Plank (3 × 20 s)	Beginner–Intermediate
**3rd Week**	5 min stretching 5 min cycling 10 min treadmill 5 min elliptical	1. Lunge 2. Seated Row 3. Incline Dumbbell Press 4. Dumbbell Shrug 5. Hammer Curl	Bicycle Crunch (3 × 20) Plank (3 × 30 s)	Intermediate
**4th Week**	5 min stretching 5 min cycling 10 min treadmill 5 min elliptical	1. Glute Kickback 2. Cable Kickback 3. Arnold Press 4. Russian Twist (with load) 5. Leg Raise	Russian Twist (3 × 20) Side Plank (3 × 30 s)	Intermediate
**5th Week**	5 min stretching 5 min cycling 10 min treadmill 5 min elliptical	1. Squat (increased load) 2. Leg Curl 3. Push Down 4. Lat Pull Down 5. Biceps Curl 6. Hip Thrust	Toe Touch Crunch (3 × 15) Mountain Climbers (3 × 30 s)	Intermediate–Advanced
**6th Week**	5 min stretching 5 min cycling 10 min treadmill 5 min elliptical	1. Deadlift (moderate load) 2. Arnold Press 3. Leg Extension 4. Seated Row 5. Dumbbell Chest Press 6. Dumbbell Lunge	Flutter Kicks (3 × 30 s) Plank + Shoulder Tap (3 × 15)	Advanced
**7th Week**	5 min stretching 5 min cycling 10 min treadmill 5 min elliptical	1. Romanian Deadlift 2. Cable Kickback 3. Barbell Hip Thrust 4. Incline Dumbbell Press 5. Side Dumbbell Raise 6. Dumbbell Shrug	V-Up (3 × 10) Leg Raise (3 × 12)	Advanced
**8th Week**	5 min stretching 5 min cycling 10 min treadmill 5 min elliptical	1. Squat 2. Lunge 3. Lat Pull Down 4. Hip Thrust 5. Biceps Curl 6. Deadlift (max load)	Plank (maximum duration) Russian Twist (1 min)	Advanced—Test Week

**Table 2:** Pre- and post-test comparisons of anthropometric and body composition variables across BMI categories.

Variable		Diabetes (a)		Underweight (b)		Normal (c)		Overweight (d)		Obese (e)		F	*p*	Post Hoc Comparison	η^2^
		n	Mean ± SS	n	Mean ± SS	n	Mean ± SS	N	Mean ± SS	n	Mean ± SS				
**BMİ**	**PRE**	8	29.06 ± 4.49	8	17.89 ± 0.50	8	22.41 ± 0.98	8	26.14 ± 0.85	8	32.90 ± 2.98	43.64	**0.000**	a > b, c	0.83
	**POST**	8	27.62 ± 3.83	8	18.73 ± 0.74	8	24.76 ± 0.92	8	24.76 ± 0.88	8	30.50 ± 3.02	32.21	**0.000**	a > b, c	0.78
**WG**	z = 2.527 *p* = **0.012**			t = −3.134, *p* = **0.017**		t = 0.574, *p* = 0.584		t = 9.924, *p* = **0.000**		t = 21.466, *p* = **0.000**					
**Cohen d**	1.797			−1.108		0.203		3.509		7.589					
**Body Weight**	**PRE**	8	77.69 ± 9.92	8	49.51 ± 2.85	8	60.04 ± 4.66	8	69.39 ± 3.14	8	91.16 ± 10.18	42.411	**0.000**	a > b, c	0.82
	**POST**	8	73.80 ± 8.82	8	52.21 ± 1.90	8	58.83 ± 4.02	8	65.54 ± 3.08	8	84.28 ± 9.74	31.322	**0.000**	a > b, c	0.78
**WG**	t = 6.546, *p* = **0.000**			t = 5.459, *p* = **0.001**		t = 2.737, *p* = **0.029**		t = 10.563, *p* = **0.000**		z = 2.521, *p* = **0.012**					
**Cohen d**	2.314			−1.930		0.968		3.734		5.220					
**Fat Mass**	**PRE**	8	41.56 ± 3.16	8	21.03 ± 3.89	8	31.30 ± 3.76	8	34.39 ± 2.93	8	43.60 ± 4.89	45.204	**0.000**	a > b, c, d	0.83
	**POST**	8	37.36 ± 1.71	8	19.64 ± 3.49	8	28.89 ± 3.71	8	30.73 ± 2.71	8	38.15 ± 3.66	45.424	**0.000**	a > b, c, d	0.83
**WG**	t = 5.991, *p* = **0.001**			t = 5.559, *p* = **0.001**		z =2.521, *p* = **0.012**		t = 11.796, *p* = **0.000**		t = 9.083, *p* = **0.000**					
**Cohen d**	2.118			1.965		2.935		4.170		3.211					
**Muscle Mass**	**PRE**	8	25.41 ± 0.96	8	32.74 ± 2.02	8	29.93 ± 3.79	8	29.14 ± 2.03	8	25.33 ± 2.68	13.056	**0.000**	a < b, c, d	0.59
	**POST**	8	29.70 ± 0.86	8	35.39 ± 1.67	8	32.60 ± 3.59	8	32.79 ± 2.73	8	29.81 ± 2.92	6.958	**0.000**	a < b, c, d	0.44
**WG**	t = 8.591, *p* = **0.000**			t = 16.192, *p* = **0.000**		t = 7.526, *p* = **0.000**		t = 11.226, *p* = **0.000**		z = 2.521, *p* = **0.012**					
**Cohen d**	−3.037			−5.275		−2.661		−3.669		−3.200					
**Visceral Fat Level**	**PRE**	8	7.88 ± 1.64	8	2.38 ± 0.92	8	3.75 ± 1.16	8	1.16 ± 6.38	8	8.00 ± 1.20	27.652	**0.000**	a > b, c	0.76
	**POST**	8	6.13 ± 1.36	8	1.50 ± 0.53	8	1.50 ± 0.53	8	0.53 ± 3.13	8	4.75 ± 0.46	32.853	**0.000**	a > b, c, d	0.79
**WG**	t = 10.693, *p* = **0.000**			z = 1.890, *p* = **0.059**		t = 6.148, *p* = **0.000**		z = −2.640, *p* = **0.008**		t = 10.370, *p* = **0.000**					
**Cohen d**	3.780			0.883		2.174		7.021		3.666					

**Abbreviations: n** indicates the number of participants, **SD** standard deviation, **PRE** pre-test (baseline), **POST** post-test, **BMI** body mass index, **WG** within-group comparison, **t** paired-samples *t*-test, **z** Wilcoxon signed-rank test, **F** F statistic obtained from one-way analysis of variance, **p** probability value, **Post hoc** post hoc comparison, **η^2^** eta squared as the effect size for between-group comparisons, **Cohen’s d** effect size for within-group changes, and group labels are defined as **a** Diabetes, **b** Underweight, **c** Normal weight, **d** Overweight, and **e** Obese.

**Table 3:** Pre- and post-test comparisons of anthropometric and body composition variables across BMI categories.

Variable		Diabetes (a)		Underweight (b)		Normal (c)		Overweight (d)		Obese (e)		F/x^2^ KW	*p*	Post Hoc Comparison	η^2^
		n	Mean ± SS	n	Mean ± SS	n	Mean ± SS	n	Mean ± SS	n	Mean ± SS				
**Myonectin**	**PRE**	8	0.76 ± 0.23	8	0.91 ± 0.45	8	0.86 ± 0.27	8	0.84 ± 0.27	8	0.81 ± 0.33	x^2^kw = 0.623	0.961	-	0.02
	**POST**	8	0.81 ± 0.19	8	0.98 ± 0.57	8	0.63 ± 0.18	8	0.66 ± 0.24	8	0.65 ± 0.17	x^2^kw = 4.617	0.329	-	0.17
**WG**	t = 0.445, *p* = 0.670			z = 0.000, *p* = 1.000		t = 2.651, *p* = **0.033**		t = 1.680, *p* = 0.093		z = 1.540, *p* = 0.123					
**Cohen d**	−0.157			−0.092		0.937		0.600		0.560					
**HIF-1α**	**PRE**	8	1.39 ± 0.56	8	1.02 ± 0.52	8	1.26 ± 0.96	8	0.94 ± 0.27	8	1.30 ± 0.77	x^2^kw = 2.544	0.637	-	0.073
	**POST**	8	1.44 ± 0.65	8	0.83 ± 0.43	8	0.95 ± 0.44	8	1.09 ± 0.46	8	1.54 ± 0.67	F = 2.656	**0.049**	a > b	0.233
**WG**	t = −0.284, *p* = 0.784			z = −1.120, *p* = 0.263		z = −0.094, *p* = 0.925		t = −1.250, *p* = 0.252		z = −0.420, *p* = 0.674					
**Cohen d**	−0.101			0.341		0.276		−0.442		−0.227					
**İrisin**	**PRE**	8	1.23 ± 0.22	8	1.08 ± 0.44	8	1.21 ± 0.33	8	1.02 ± 0.34	8	1.09 ± 0.22	F = 0.608	0.660	-	0.06
	**POST**	8	2.14 ± 0.31	8	1.63 ± 0.51	8	1.81 ± 0.46	8	1.57 ± 0.40	8	1.92 ± 0.31	F = 2.581	**0.050**	a > b, c	0.22
**WG**	t = 6.966, *p* = **0.000**			t = 4.712, *p* = **0.002**		t = −3.435, *p* = **0.011**		z = −2.521, *p* = **0.012**		t = −8.857, *p* = **0.000**					
**Cohen d**	−2.463			−1.666		−1.214		−4.802		−3.036					
**İnsulin**	**PRE**	8	1.11 ± 0.57	8	0.50 ± 0.40	8	0.53 ± 0.36	8	0.36 ± 0.18	8	0.62 ± 0.51	x^2^kw = 12.773	**0.012**	a > b, c, d, e	0.29
	**POST**	8	0.66 ± 0.28	8	0.32 ± 0.19	8	0.45 ± 0.28	8	0.19 ± 0.06	8	0.43 ± 0.43	x^2^kw = 15.043	**0.005**	a > b, c, d	0.26
**WG**	z = −2.521, *p* = **0.012**			t = 1.739, *p* = 0.126		z = −4.637, *p* = **0.000**		z = −2.380, *p* = **0.017**		z = −2.380, *p* = **0.017**					
**Cohen d**	1.079			0.615		0.521		1.042		1.447					
**Glucose**	**PRE**	8	109.13 ± 19.22	8	80.63 ± 7.44	8	84.13 ± 9.23	8	85.38 ± 10.86	8	93.50 ± 15.11	F = 6.075	**0.001**	a > b, c, d, e	0.41
	**POST**	8	89.13 ± 8.66	8	76.38 ± 5.85	8	75.50 ± 4.14	8	77.75 ± 4.50	8	87.75 ± 12.52	F = 5.713	**0.001**	a > b, c, d	0.39
**WG**	t = 4.632, *p* = **0.002**			z = −1.682, *p* = 0.092		t = 2.799, *p* = **0.027**		t = 3.077, *p* = **0.018**		t = 4.403, *p* = **0.003**					
**Cohen d**	1.638			0.553		0.989		1.088		1.557					

**Abbreviations:** Values are presented as mean ± standard deviation (SD), and n indicates the number of participants. PRE and POST denote pre-test (baseline) and post-test measurements, respectively. WG indicates within-group (pre–post) comparisons, analyzed using the paired-samples *t*-test (t) or Wilcoxon signed-rank test (z), as appropriate. Between-group differences were assessed using one-way analysis of variance (F) or the Kruskal–Wallis test (χ^2^KW). *p* < 0.05 was considered statistically significant. Post hoc comparisons identify significant between-group differences (e.g., a > b, c). η^2^ represents effect size for between-group comparisons, and Cohen’s d represents effect size for within-group changes. Group labels are a = Diabetes, b = Underweight, c = Normal weight, d = Overweight, and e = Obese.

## Data Availability

The datasets generated or analyzed during the current study are available from the corresponding author upon reasonable request.
